# Biomechanical stability of tape augmentation for anterior talofibular ligament (ATFL) repair compared to the native ATFL

**DOI:** 10.1007/s00167-016-4048-7

**Published:** 2016-02-15

**Authors:** M. Willegger, E. Benca, L. Hirtler, K. Hradecky, J. Holinka, R. Windhager, R. Schuh

**Affiliations:** Department of Orthopaedics, Medical University of Vienna, Waehringer Guertel 18-20, 1090 Vienna, Austria; Institute of Anatomy, Medical University of Vienna, Vienna, Austria

**Keywords:** Anterior talofibular ligament reconstruction, Lateral ankle instability, Tape augmentation, Bone mineral density, Biomechanical stability

## Abstract

**Purpose:**

Current methods of anterior talofibular ligament (ATFL) reconstruction fail to restore the stability of the native ATFL. Therefore, augmented anatomic ATFL reconstruction gained popularity in patients with attenuated tissue and additional stress on the lateral ankle ligament complex. The aim of the present study was to evaluate the biomechanical stability of the InternalBrace^®^ (Arthrex Inc., Naples, FL, USA), a tape augmentation designed to augment the traditional Broström procedure.

**Methods:**

Twelve (12) fresh-frozen human anatomic lower leg specimens were randomized into two groups: a native ATFL (ATFL) and a tape augmentation group (IB). Dual-energy X-ray absorptiometry (DEXA) scans were carried out to determine bone mineral density (BMD) of the specimens. The ligaments were stressed by internally rotating the tibia against the inverted fixated hindfoot. Torque at failure (Nm) and angle at failure (°) were recorded.

**Results:**

The ATFL group failed at an angle of 33 ± 10°. In the IB group, construct failure occurred at an angle of 46 ± 16°. Failure torque reached 8.3 ± 4.5 Nm in the ATFL group, whereas the IB group achieved 11.2 ± 7.1 Nm. There was no correlation between angle at ATFL or IB construct failure or torque at failure, respectively, and BMD for both groups.

**Conclusion:**

This study reveals that tape augmentation for ATFL reconstruction shows similar biomechanical stability compared to an intact native ATFL in terms of torque at failure and angle at failure. BMD did not influence the construct stability. Tape augmentation proved an enhanced initial stability in ATFL reconstruction which may allow for an accelerated rehabilitation process.

**Level of evidence:**

II.

## Introduction

Anatomic repair of the anterior talofibular ligament (ATFL) as described by Broström [5] is considered the standard surgical treatment for patients with chronic lateral ankle instability [13, 38]. Despite adequate clinical results, recent biomechanical studies revealed that current methods of reconstruction fail to fully restore the biomechanical strength of the native ATFL [6, 16, 17, 19, 27, 36, 37]. Mechanical instability and functional instability represent major complications of ATFL reconstruction [12, 20, 33]. High stresses acting on the reconstructed ATFL may lead to ATFL elongation during post-operative rehabilitation with consecutive instability of the ankle joint [24]. In particular, patients with pre-existing pathologic conditions that strain the lateral ankle ligaments (i.e. cavovarus deformity, joint hyperlaxity and overweight) require substantial primary stability of the construct in order to withstand the supplemental applied stress and to prevent early failure [26, 31, 33]. Additionally, patients with attenuated tissue, previously failed ATFL reconstruction and professional athletes are in danger to tear the reconstructed ATFL [13, 30, 31].

Therefore, augmented anatomic ATFL reconstruction has been endorsed for patients with exposure for ATFL reconstruction failure [33]. Various techniques have been proposed to enhance the initial construct stability of anatomic ATFL reconstruction, using suture anchor techniques [29] or requiring free tendon grafts [11, 33]. Due to the potential risk of donor-site morbidity after graft harvesting and the still inferior biomechanical results of suture anchor techniques compared to the native ATFL, there is a demand for less invasive and biomechanically more stable reconstructions. Hence, recent attention has been focussed on tape augmentation to improve primary stability of lateral ankle ligament repair [7, 34, 36]. It operates as internal additional reinforcement of an anatomic ATFL reconstruction in order to enhance the initial construct stability and to prevent early failure or elongation. Tape augmentation can be performed by open surgery or arthroscopically assisted surgery.

Post-operative early range of motion rehabilitation has been shown to have superior outcome regarding plantar flexion strength, mechanical stability of the ankle and return to sports activities, compared to cast immobilization [20, 21]. Enhanced primary stability of ATFL reconstruction could allow for an accelerated rehabilitation protocol without the need for cast immobilization.

However, little is known about the biomechanical characteristics of tape augmentation [34, 36]. Additionally, the influence of bone mineral density on the stability of a tape augmentation device has not been examined yet.

In the present study, the stability of the InternalBrace^®^ (Arthrex Inc., Naples, FL, USA), a tape augmentation designed to augment a traditional Broström procedure utilizing BioComposite SwiveLock^®^ (Arthrex Inc., Naples, FL, USA) and FiberTape^®^ (Arthrex Inc., Naples, FL, USA), has been evaluated. We hypothesized that tape augmentation would show similar biomechanical stability compared to an intact native ATFL at time zero. Further, correlation between BMD and construct stability has been analysed.

## Materials and methods

Twelve (12) fresh-frozen human anatomic ankle specimens (mean age 78.2 ± 11.6 years; range 60–92 years; six male and six female) were used for data collection of this study. All specimens were screened for evidence of ankle instability, hyperlaxity, fracture and previous ankle or hindfoot surgery. Prior to biomechanical testing, dual-energy X-ray absorptiometry (DEXA) scans were carried out in the calcaneus to determine bone mineral density (BMD) of the specimens. All measurements were taken with Lunar, Prodigy series X-ray, GE Medical Systems (GE Healthcare Europe GmbH, Vienna). This system provides precise data on soft tissue and bone composition including bone mineral density (BMD), lean and fat tissue mass and percentage of fat. Repeatability of DEXA measurements has been excellent for bone mineral density using Lunar Prodigy densitometer in prior studies [28].

The specimens were stored at –80 °C and thawed at room temperature only 48 h before use in order to prevent a possible tissue dehydration, which can affect their mechanical properties [40, 41].

Following inspection of the specimens, all ankles proved validity for the study and thereafter were randomly assigned to two different groups with six specimens in each group. Age distribution and BMD were similar among the groups. The specimens of the first group served as the native ATFL group (ATFL) and the second group served as tape augmentation (InternalBrace^®^, Arthrex Inc., Naples, FL, USA) group (IB).

### Specimen preparation

Each specimen was transected at the mid-tibia approximately 25 cm above the ankle joint. Soft tissue was removed, and the proximal aspect of the tibia was rigidly embedded in a custom-made steel cup with Wood’s metal [22]. The fibula was then drilled to the tibia with a conventional 4.0-mm screw in order to prevent tibiofibular movement during biomechanical testing [34, 40, 41].

For biomechanical testing of the ATFL stability (ATFL group), ankle specimens were left intact after reassurance for an unimpaired ATFL. Anterior drawer test and talar tilt test were conducted to check for intact ligaments.

**Fig. 1:**
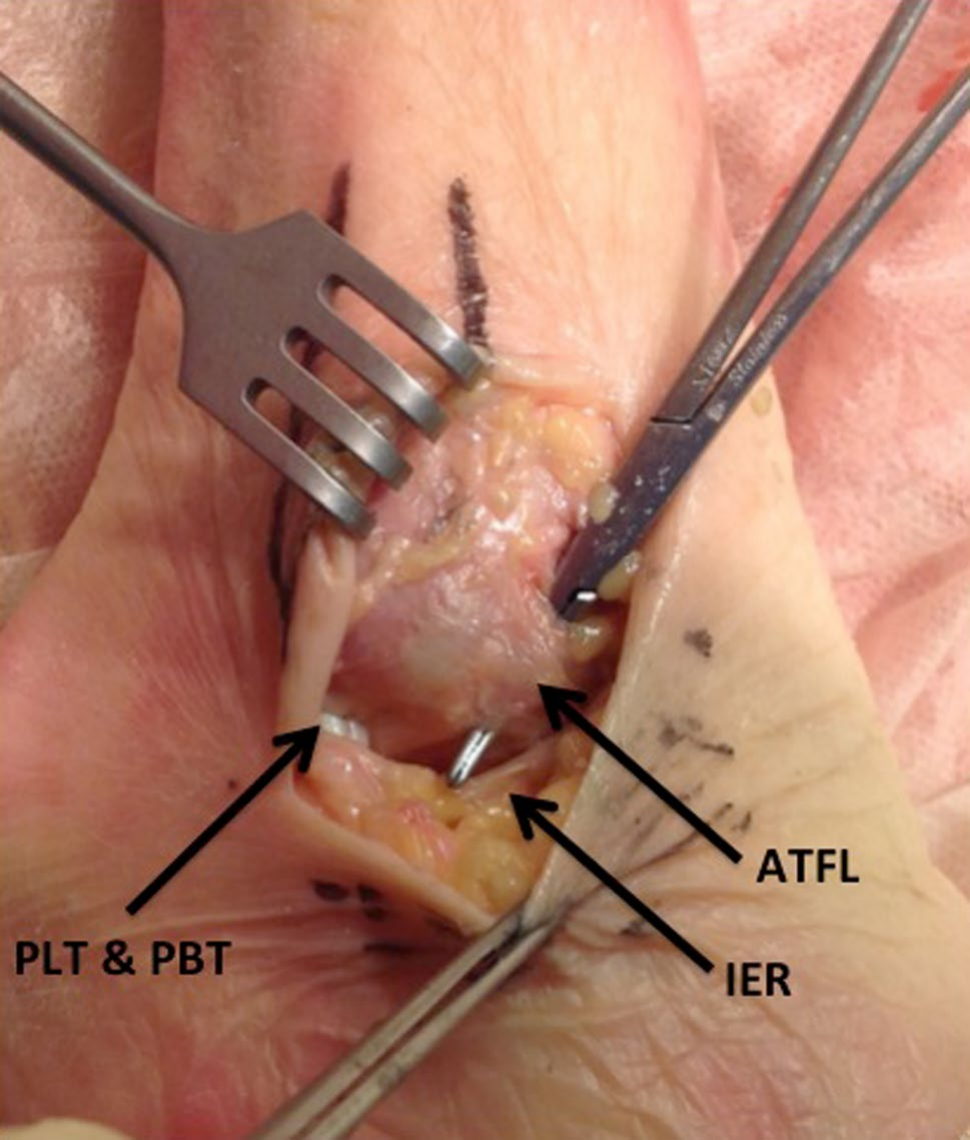
Surgical exposure before dissection of the ATFL in a right ankle specimen from the IB group. A curved haemostat is placed under the anterior talofibular ligament (ATFL). *IER* inferior extensor retinaculum, *PLT* peroneus longus tendon, *PBT* peroneus brevis tendon

In specimens which were performed with tape augmentation (IB group), a J-shaped incision was carried out just anterior to the fibula to allow easy exposure of the anterolateral capsule and ATFL and calcaneofibular ligament (CFL) [1]. The incision extended from the distal tip of the fibula along its anterior margin proximally to the level of the ankle mortise. Dissection was taken down to the fibular periosteum. The joint capsule was incised in line with the skin incision and just distal to the leading edge of the fibula. The ATFL and CFL were directly inspected, and all specimens had intact ligaments. Subsequently, a curved haemostat was placed within the lateral ankle joint and passed under the lateral capsule and the ATFL, exiting just anterior to the peroneal tendon sheath (Fig. 1). The ATFL was meticulously divided in mid-substance with a scalpel. The anterior drawer test was conducted in order to confirm the creation of anterior instability of the ankle.

The biomechanical stability of the augmentation construct (IB group) was tested by application of the InternalBrace^®^ at the insertion and origin of the ATFL [1, 39]. A hole was drilled with the 2.7-mm drill in the fibula, angled slightly proximally, in line with the lateral border of the foot. Afterwards, the hole was taped with a 3.5-mm tape to breach the fibular cortex. The 3.5-mm SwiveLock^®^ was loaded with FiberTape^®^ and placed into the fibular hole by turning the driver clockwise. Then the 3.4-mm drill was drilled into the lateral aspect of the talus in line with the superior ATFL directed 45° posteromedially with respect to the lateral border of the foot. The talar tunnel was taped with the 4.75-mm SwiveLock^®^ Tape. Range of motion was checked before the second anchor was inserted. Both limbs of the FiberTape^®^ were passed through the eyelet of the 4.75-mm SwiveLock^®^, and the anchor was inserted with the foot placed in a slightly plantar-flexed position with 5° of eversion. To avoid overtensioning, a small curved haemostat was placed between the FiberTape^®^ and talus while inserting the SwiveLock^®^ [37]. All dissections and repairs were performed by a single experienced foot and ankle surgeon.

### Biomechanical testing

**Fig. 2:**
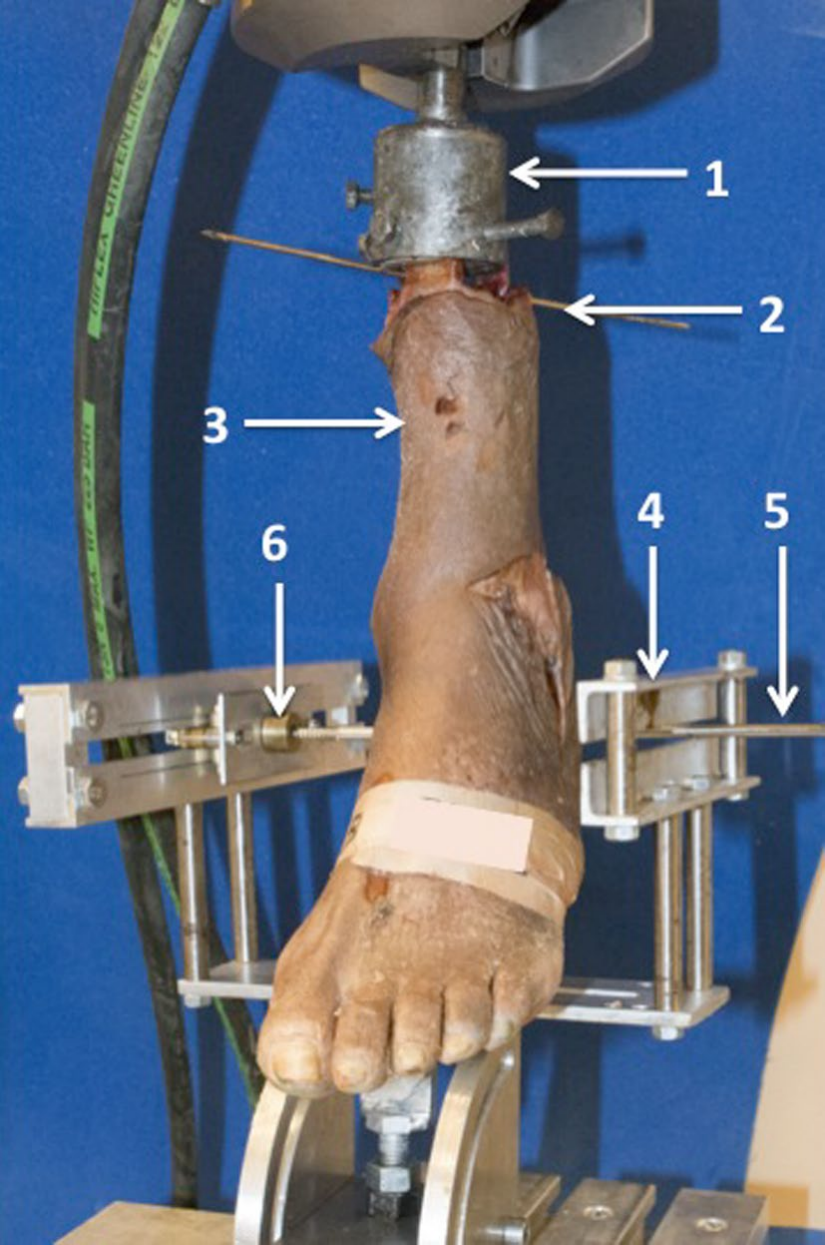
Biomechanical test set-up: Wood’s metal and a custom-made steel cup (*1*) were used for proximal fixation. A Kirschner wire (*2*) prevented relative tibiofibular movement. The lower leg specimen (*3*) was mounted into the testing platform (*4*) using a Kirschner wire (*5*) drilled through the calcaneus and locked into the guide block (*6*) of the mounting platform

The mechanical testing for this study was performed with an 858 Mini Bionix^®^ (MTS^®^ Systems Corporation, Eden Prairie, MN, USA) and a specially designed mounting platform. The 858 Mini Bionix^®^ is a servo-hydraulic test frame, consisting of a loading frame (MTS^®^ 858, Eden Prairie, MN, USA) with a stroke main actuator driven by a hydraulic pump unit (MTS^®^ 505.11 silent flow, Eden Prairie, MN, USA). The lower leg specimen was mounted in the cylindrical clamp of the 858 Mini Bionix^®^. All specimens were positioned with their mechanical tibial axis coinciding with the rotational axis of the 858 Mini Bionix^®^ by use of a fixed laser beam. Furthermore, the specimens were placed in 20° of plantar flexion and 15° of hindfoot inversion to simulate the kinematics during ankle sprain [3, 25]. The specially designed mounting platform enables fixation of the calcaneus with a 4.5-mm Steinmann pin. The pin was drilled through the calcaneus, posterior to the longitudinal axis of the tibia. Additionally, poly(methyl methacrylate) cement (PMMA) was used to rigidly fixate the Steinmann pin within the calcaneus in order to prevent loosening of the pin with subsequent inaccuracy in measurements. The Steinmann pin was then locked in a guide block attached to the platform. The platform and the load frame allow accurate positioning of the proximal and distal fixation of the ankle specimen [34, 40, 41] (Fig. 2).

**Fig. 3:**
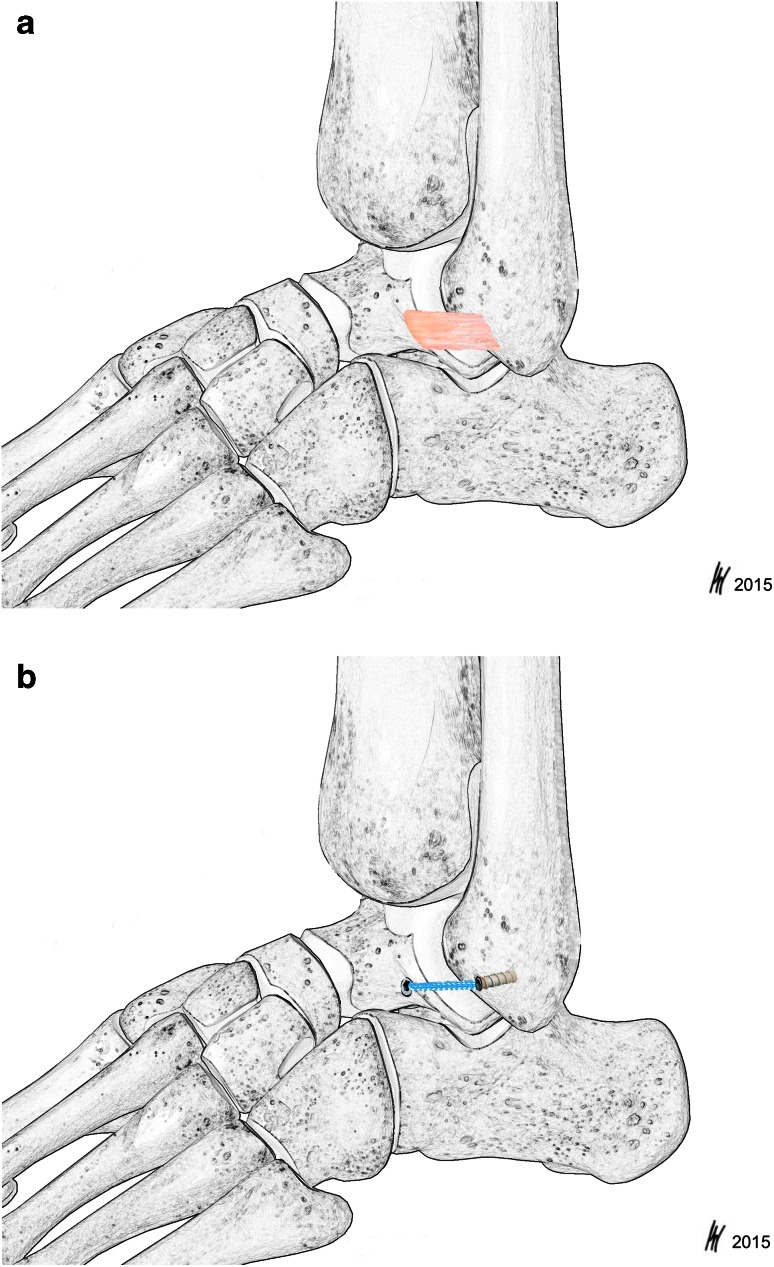
**a** and **b** Schematic drawing of the native ATFL (**a**) (group ATFL) and the InternalBrace^®^ (Arthrex Inc., Naples, FL, USA) tape augmentation (**b**) (group IB)

In order to simulate stresses in the lateral ankle ligaments during in vivo ankle sprain, torsion was applied on specimens by internal rotation of the tibia against the fixated hindfoot from 0° to 50°, followed by re-relaxation to 0°. The guide block of the platform enables anterior-to-posterior motion of the hindfoot during internal rotation allowing anterior translation of the talus. Hence, the ATFL can be maximally stressed. The first test series was carried out on the healthy specimens with intact ATFL and CFL (ATFL group) (Fig. 3a), followed by the specimens with transected ATFL and InternalBrace^®^ augmentation (IB group) (Fig. 3b). The torque (Nm) required to resist the internal rotation as well as the rotary displacement (°) of the load frame was recorded at a measuring frequency of 100 Hz. The procedure was stopped at the maximum of 50° internal rotation followed by unloading.

The measurement transducer for the angular displacement and the torque is integrated into the 858 Mini Bionix II^®^ testing system. The accuracy for the angular displacement transducer is <0.3 and <0.5 % for the torque transducer, respectively.

The study was approved by the Institutional Review Board (EK 1895/2013) of the Medical University of Vienna.

### Statistical analysis

All analyses were performed using SPSS 20.0 for Windows (SPSS Inc, Chicago, IL, USA), and the level of significance was set at *p* < 0.05. All data showed a normal distribution in Kolmogorov–Smirnov test. Intergroup differences in age, BMD, failure angle and failure torque were analysed using independent *t* test.

For *t* tests that demonstrated a statistically significant difference, a post hoc Tukey’s honestly significant difference test was conducted to assess the location of the means that were statistically significant between the groups. Correlation was analysed with Pearson correlation coefficient. A post hoc power analysis was performed with G*Power 3.1 for MAC OS X (http://www.gpower.hhu.de).

## Results

Both groups of specimens did not differ by age, sex or bone mineral density (BMD) (Table 1). The biomechanical testing results are summarized in Table 2. Torque at failure reached 8.3 ± 4.5 Nm in the ATFL group, whereas the IB group achieved 11.2 ± 7.1 Nm (n.s.). The intact ATFL group failed at an angle of 33 ± 10° of internal rotation. In the IB group, construct failure occurred at an angle of 46 ± 16° (n.s.).

**Table 1:** Descriptive data of the tested ankle specimens

Group	Intact ATFL (*n* = 6)	Tape augmentation (InternalBrace) (*n* = 6)	*p* values
Age (years)	76.6 ± 12.8	79.6 ± 11.2	n.s.
BMD (g/cm^2^)	0.46 ± 0.18	0.44 ± 0.16	n.s.
Sex (male/female)	3 m/3 f	3 m/3 f	n.s.
Side (left/right)	2 l/4 r	4 l/2 r	n.s.

**Table 2:** Mean values and standard deviations of maximal tolerated torque to failure and failure angle at maximum tolerated torque to failure

Group	Intact ATFL (*n* = 6)	Tape augmentation (InternalBrace) (*n* = 6)	*p* values
Max. failure torque (Nm)	8.3 ± 4.5	11.2 ± 7.1	n.s.
Failure angle (°)	33 ± 10	46 ± 16	n.s.

Mean BMD in the native ATFL group was 0.46 ± 0.18 and 0.44 ± 0.16 g/cm^2^ in the IB group (n.s.). There was no correlation between angle at ATFL or IB construct failure or torque at failure, respectively, and BMD for both groups.

Assessment of the mode of failure after mechanical testing revealed that the most common mode of failure in the ATFL group was rupture at the mid-portion of the ligament (*n* = 5 out of 6). Two of those specimens had an additional partial rupture of the calcaneofibular ligament (CFL). One specimen failed at the fibular origin of the ATFL. In the IB group, five constructs failed due to pull-out of the talar anchor and one anchor pull-out occured at the fibula. No correlation between BMD and mode of failure could be observed.

## Discussion

The most important finding of the study was a similar initial construct stability for the InternalBrace^®^ compared to the intact native ATFL. Various ATFL reconstruction techniques and modifications have been proposed, aiming to improve construct stability [2, 8, 10, 15, 18, 23, 29]. Nevertheless, recent biomechanical studies showed that current methods of anatomic ATFL reconstruction do not restore the strength of the native ATFL [36, 37]. Patients with additional strains on the lateral ankle ligaments due to hindfoot cavovarus deformity, poor tissue quality, obesity or professional athletes require initial stability after anatomic ATFL reconstruction, preventing elongation or failure of the reconstruction and allowing early mobilization. Therefore, recently augmented ATFL reconstruction was introduced. This study aimed to evaluate the biomechanical stability of tape augmentation compared to the intact native ATFL.

Both groups showed comparable means in terms of torque and angle at failure during biomechanical testing. Additionally, we found no correlation of bone mineral density and torque or angle at failure between the two groups. Mode of failure assessment showed rupture at the mid-portion of the ATFL as the most common failure in the native ATFL group. In the tape augmentation group, talar anchor pull-out was the most common mode of failure.

The torque at failure in the IB group reached 11.2 ± 7.1 Nm compared to 8.3 ± 4.5 Nm in the ATFL group. The intact ATFL constructs failed at a mean angle of 33 ± 10° of internal rotation, and the IB group failed at a mean angle of 46 ± 16°. Both groups of specimens showed no difference in bone mineral density with 0.46 ± 0.18 g/cm^2^ in the ATFL and 0.44 ± 0.16 g/cm^2^ in the IB group.

Numerous authors focussed on the biomechanical characteristics of suture anchor repair in anatomic reconstruction techniques compared to the native ATFL. They revealed that the reconstructed ATFL has inferior biomechanical stability in terms of force resistance than the native ATFL [36, 37].

Viens et al. [36] investigated biomechanical characteristics of tape augmentation, the intact ATFL and an augmented Broström repair. A load to failure test was carried out stressing the lateral ligaments by axial load application through the fibula. The ultimate load to failure of the tape augmentation group was statistically significantly higher than in the intact ATFL group. However, there was no statistically significant difference between the intact ATFL and the Broström repair with tape augmentation. The authors did not comment on the finding that the Broström repair with augmentation showed less strength than the stand-alone augmentation. Nevertheless, they observed higher biomechanical stability of the suture tape augmentation in relation to the native ATFL which corresponds to our findings in terms of tolerated torque and angle at failure. Schuh et al. [34] showed statistically superior performance concerning angle at failure as well as torque at failure for an InternalBrace^®^ with Broström reconstruction compared to a suture anchor fixated Broström and a traditional Broström repair. BMD was analysed and did not influence the construct stability in the suture anchor repair groups. Another study by Clanton et al. [9] compared an anatomic ATFL reconstruction with semitendinosus allograft to an intact ATFL in a biomechanical load to failure setting. Ultimate load to failure and stiffness of the allograft were not significantly different from the native ATFL. Nevertheless, clinical studies for allograft use in ATFL reconstruction are sparse with small retrospective case series, and the potential risks of disease transmission or rejection of the graft by the host limit its widespread clinical use [14, 32].

Kirk et al. [24] postulated the need for post-operative protected dorsi- and plantar flexion of the ankle after ATFL repair. In a cyclic loading model, they tested elongation of the unprotected repaired ATFL, the protected repaired ATFL, the protected intact ATFL and protected sectioned ATFL. Initial elongation was significantly higher in the unprotected repaired group than in the protected groups. Avoidance of ligament elongation could be provided by the internal protection of the reconstructed ATFL by tape augmentation.

Different types of modes of failure in ATFL rupture and ATFL reconstruction failure have been described. In our test series, talar anchor pull-out was the most common mode of failure in the IB group. Ligament rupture in mid-substance was the most frequent mode of failure in the native ATFL group which confirms our test set-up to be effective in simulation of in vivo ankle sprain kinematics. Anchor pull-out is the most common mode of failure in biomechanical studies testing suture anchor reconstruction [16, 17, 34, 36, 37].

These findings might be explained by high donor age and poor bone mineral density of the tested specimens. In rotator cuff repair, low bone mineral density has been shown to have a significant impact on anchor failure [4, 35]. There are a few biomechanical studies focussing on lateral ankle ligament reconstruction with suture anchors investigating the influence of BMD on construct stability [6, 16, 17, 34, 36, 37]. Therefore, DEXA scans were performed in advance to assess the bone mineral density of the specimens, in order to question the influence of BMD on the mode of failure, torque at failure and angle at failure. In our study, we could not find any correlation between mode of failure and BMD.

There are some potential limitations associated with this study. First, we did not perform a matched-pair study. To compensate for this potential drawback, we determined bone mineral density prior to mechanical testing in order to prove equality of the two specimen groups in addition to a similar age distribution among groups. Nevertheless, even in matched-pair studies the structure and the quality of the ligamentous tissue can only be assessed by direct inspection or by stressing the lateral ligaments with talar tilt and anterior drawer test, which we performed meticulously. Second, the number of tested specimens was relatively low with 12 tested specimens, which is common in biomechanical studies due to unavailability of human anatomic specimens. Third, as in every biomechanical test set-up, we tried to simulate in vivo conditions in a laboratory setting which might not represent in vivo stresses acting on the ATFL. However, as we aimed to evaluate the biomechanical characteristics of the intact ATFL and the InternalBrace^®^, we applied torsion until failure in a plantar flexed and adducted foot.

In case of a sudden inversion injury of the ankle while the reconstructed ligament is not completely healed, re-rupture could occur [26, 31, 33]. As prior biomechanical studies demonstrated, the proposed commonly used reconstructions showed a decreased stability compared to the native ATFL [36, 37]. Therefore, the additional protective strength applied with tape augmentation to a standard Broström repair could allow for an earlier and more active remobilization regimen. Less strain would act on the Broström reconstruction, and ligament healing could proceed without the risk of ATFL elongation with consecutive lateral ankle instability. However, these potential biomechanical advantages have to be proven clinically.

## Conclusion

The present biomechanical study reveals that tape augmentation for ATFL reconstruction shows similar biomechanical stability compared to an intact native ATFL in terms of torque at failure and angle at failure. BMD did not seem to affect ATFL or IB construct failure.
